# Pharmacotherapeutic Considerations in the Treatment of Nontuberculous Mycobacterial Infections: A Primer for Clinicians

**DOI:** 10.1093/ofid/ofae128

**Published:** 2024-03-15

**Authors:** Christo Cimino, Christina G Rivera, Jeffrey C Pearson, Benjamin Colton, Douglas Slain, Monica V Mahoney

**Affiliations:** Department of Pharmaceutical Services, Vanderbilt University Medical Center, Nashville, Tennessee, USA; Department of Pharmacy, Mayo Clinic, Rochester, Minnesota, USA; Department of Pharmacy, Brigham and Women's Hospital, Boston, Massachusetts, USA; Pharmacy Department, National Institutes of Health Clinical Center, Bethesda, Maryland, USA; Department of Clinical Pharmacy, School of Pharmacy and Section of Infectious Diseases, School of Medicine, West Virginia University, Morgantown, West Virginia, USA; Department of Pharmacy, Beth Israel Deaconess Medical Center, Boston, Massachusetts, USA

**Keywords:** antimycobacterial, nontuberculous *Mycobacterium*, pharmacist, pharmacotherapy

## Abstract

Nontuberculous mycobacteria (NTM) can cause a variety of infections, including serious pulmonary disease. Treatment encompasses polypharmacy, with a targeted regimen of 2–5 active medications, depending on site of infection, species, and clinical characteristics. Medications may include oral, intravenous, and inhalational routes. Medication acquisition can be challenging for numerous reasons, including investigational status, limited distribution models, and insurance prior authorization. Additionally, monitoring and managing adverse reactions and drug interactions is a unique skill set. While NTM is primarily medically managed, clinicians may not be familiar with the intricacies of medication selection, procurement, and monitoring. This review offers insights into the pharmacotherapeutic considerations of this highly complex disease state, including regimen design, medication acquisition, safety monitoring, relevant drug–drug interactions, and adverse drug reactions.

Nontuberculous mycobacteria (NTM) are found naturally in the environment, especially in soil and water, with distribution varying by region, depending on climate, moisture, and other environmental conditions [1–3]. NTM comprise ∼200 individual species of mycobacteria, excluding *Mycobacterium tuberculosis* and *Mycobacterium leprae* [1, 2, 4]. The prevalence of NTM infection appears to be increasing, at least in part due to improved diagnostics [5, 6], with *Mycobacterium avium* complex (MAC) representing the most common pathogen [1, 6, 7]. Diagnosis requires a combination of clinical, microbiological, and radiographic information; isolation of NTM alone does not establish active infection [1]. NTM are categorized as slow growing (eg, MAC, *M. kansasii, M. marinum*) or rapid growing (eg, *M. chelonae, M. fortuitum, M. abscessus*) based on their growth rate on culture media, taking >7 or <7 days for growth, respectively [1, 3–5]. It is critical to distinguish species, and even subspecies, given the heterogeneous nature and implications for management [1, 2, 4]. Chronic pulmonary disease is reported in ∼70%–90% of NTM cases, but infection involving other sites, including soft tissue and bone, is also possible [1, 4, 5]. Disseminated disease is generally seen in the severely immunosuppressed [2]. In addition, *M. chimaera* disseminated disease has been linked to contaminated heater–cooler units used during cardiac surgery [2].

Interdisciplinary management of NTM infection is key, given the complex pharmacologic and monitoring strategies, varied drug procurement, prolonged combination therapies, and oftentimes difficult-to-tolerate agents [8]. However, given the relative infrequency of NTM infections, clinicians may feel underprepared to manage this disease. The aim of this review was to provide insights into the pharmacotherapeutics of this highly complex disease, including regimen considerations, medication acquisition, therapeutic drug monitoring (TDM), relevant drug–drug interactions, and adverse drug reactions (ADRs). Emerging therapeutic options are beyond the scope of this manuscript but have been reviewed elsewhere [9]. Given global variability in the acquisition and availability of certain NTM anti-infectives, the scope of this document will be most applicable to care in the United States.

##  

### Designing an Antimycobacterial Regimen

Treatment of NTM requires long courses of therapy lasting several months to years. In severe respiratory disease, treatment is generally continued for at least 12 months following sputum culture conversion from positive to negative growth. A duration of 2–6 months may be adequate for soft tissue infections, with 6–12 months often recommended for musculoskeletal infections [2]. Targeted regimens generally consist of 2–5 active medications, depending on clinical and microbiologic factors [10]. However, clinical data informing treatment strategies for NTM are mostly limited to observational studies, with few randomized controlled trials available. Thus, contemporary national guideline recommendations are limited to pulmonary infections caused by select species [10]. Given these limitations, treatment is often individualized based on patient-specific factors, and adherence to guideline recommendations is as low as 13% [1, 3, 11].

Antimicrobial susceptibility testing (AST) can be useful in designing NTM regimens, keeping important caveats in mind. First, NTM AST should be performed only for species associated with clinical disease [12, 13]. Second, correlation of clinical response to in vitro susceptibility patterns is established only for select species and drug pairs. For MAC isolates, clinical correlation is limited to the macrolides and amikacin (with inhaled and intravenous specific break points) [13, 14]. Clarithromycin is the class drug used for macrolide susceptibility testing of MAC. Azithromycin is the preferred macrolide for treatment of macrolide-susceptible MAC pulmonary disease, its susceptibility inferred from clarithromycin testing [10]. The minimum inhibitory concentration (MIC) data for ethambutol, rifampin, and rifabutin have shown poor correlation with clinical response for MAC. Though these drugs are often used in the treatment of MAC, susceptibility testing is not routinely recommended because a high or a low MIC value does not determine whether these drugs are useful in treatment regimens [15]. *Mycobacterium kansasii* treatment failure has been associated with rifampin resistance, but ethambutol (EMB) and isoniazid (INH) minimum inhibitory concentrations do not correlate well with clinical response [12, 13]. Susceptibility testing for other antimicrobials is recommended in the presence of macrolide resistance (for MAC) and rifampin resistance (for *M. kansasii*) [15]. An AST panel for a slow-growing NTM may consist of amikacin, ciprofloxacin, clarithromycin, clofazimine, doxycycline, linezolid, minocycline, moxifloxacin, rifabutin, rifampin, streptomycin, and trimethoprim-sulfamethoxazole [15].

For rapidly growing mycobacteria, more comprehensive AST is performed “to guide rather than dictate therapy” [12, 13, 15]. A wider panel may include amikacin, cefoxitin, clarithromycin, ciprofloxacin, clofazimine, doxycycline (or minocycline), imipenem, linezolid, moxifloxacin, tigecycline, tobramycin, and trimethoprim-sulfamethoxazole [15]. An example susceptibility report for a rapid-growing NTM is available in Supplementary Figure 1. *M. fortuitum, M. abscessus* subspecies *abscessus, M. abscessus* subspecies *bolletii,* and *M. smegmatis* isolates should be tested for inducible macrolide resistance due to the erythromycin ribosomal methylase *erm(*41*)* gene by incubation with subinhibitory concentrations of clarithromycin for 14 days [12, 15–17]. The *erm(*41*)* gene transfers 1–2 methyl groups to an adenine in the peptidyl region of 23S rRNA, preventing macrolide binding. The presence of either an *erm(*41*)* 274-bp deletion or *erm(*41*)* T28C point mutation independently indicates reversion to macrolide susceptibility, which is common for *M. abscessus* subspecies *massiliense* [18]. Azithromycin susceptibility may be inferred from clarithromycin for rapid-growing NTM as well [12]. While doxycycline or minocycline susceptibility testing may be performed for rapidly growing mycobacteria, their clinical role for the treatment of *M. abscessus* is questionable [19]. Due to the level of expertise needed and technical challenges associated with mycobacterial AST, this is typically performed at a referral microbiology laboratory. Several microbiology laboratories can perform NTM susceptibility testing such as the National Jewish Hospital, University of Texas at Tyler Health Center, and Mayo Clinic [20–27]. Waiting for AST may not be possible for disseminated and/or severe infections. Thus, empiric therapy based on the available information on mycobacterial organism susceptibility patterns is recommended until AST results are available.

Outpatient parenteral antimicrobial therapy (OPAT) is a key component in the multimodal management of NTM infections, particularly those with cavitary MAC lung disease and those with multidrug-resistant organisms such as *M. abscessus* [10, 28]. Infectious diseases–trained clinical pharmacists are well positioned to assist prescribers in the management of patients requiring OPAT for NTM, and the need for their services has expanded in the United States [29, 30]. Prospective serial laboratory and safety monitoring is of paramount importance, as duration-dependent toxicities are well described with several agents used for NTM treatment [28]. Recommended monitoring parameters are summarized in Table 1. In many instances, data supporting the frequency of laboratory monitoring are limited to low-quality evidence or expert opinion [28]. Thus, monitoring schedules should be individualized based on patient-specific factors such as severity of infection, duration of therapy, and presence of baseline organ dysfunction and/or drug interactions.

**Table 1.** Recommended Safety Monitoring Schedule for Commonly Used NTM Therapies

Antimicrobial With Typical NTM Dosing	CBC-Diff	BMP	Serum Levels	Hepatic Panel	ECG	Audiogram	Vision Exam	Comments
Amikacin (IV) [9, 10, 15, 31] 15 mg/kg daily or 5 times/wk 15–25 mg/kg 3 times/wk	Weekly	Twice weekly	Weekly trough, peak with initial dose and following dose changes			Baseline, consider monthly		Adjust dosing interval for renal dysfunction; dose using adjusted body weight for obese Consider dose adjustments based on TDM Peak: 35–45 mcg/mL (15 mg/kg) Peak: 65–80 mcg/mL (25 mg/kg) Trough: undetectable
Amikacin (inhaled, parenteral formulation) [10, 15] 250–500 mg/d		Monthly						Monitor renal function more closely in those with known or suspected renal insufficiency
Amikacin (inhaled liposomal) [9, 10, 15, 32] 590 mg daily		Monthly						Monitor renal function more closely in those with known or suspected renal insufficiency
Bedaquiline (oral) [9, 15, 33] Induction: 400 mg daily for 2 wk Maintenance: 200 mg 3 times/wk		Baseline		Baseline, monthly	Baseline, at 2, 12, and 24 wk			Close ECG monitoring with concomitant QTc-prolonging therapy
Cefoxitin (IV) [9, 10, 15, 28, 34] 2–4 g every 8–12 h (max 12 gm/d)	Weekly	Weekly		Weekly				May interfere with serum creatinine determination via Jaffé Reaction; delay lab monitoring at least 2 h after cefoxitin administration Adjust dose for renal dysfunction
Clofazimine (oral) [9, 10, 15, 31, 35] 100 mg daily				Baseline, monthly	Baseline, at 2, 12, and 24 wk			May consider TDM when indicated Peak (2 h): 0.5–2 mcg/mL Close ECG monitoring with concomitant QTc-prolonging therapy
Ethambutol (oral) [9, 10, 15, 20, 31, 36] 15–20 mg/kg daily 25 mg/kg 3 times/wk	Consider monthly	Baseline, monthly		Consider monthly			Baseline, every 3 mo (Ishihara test, fundoscopic optic exam)	May consider TDM when indicated (reference range per lab report) Dose on lean body weight Consider increasing interval for renal dysfunction
Fluoroquinolones [9, 10, 15, 28] Ciprofloxacin (IV, oral) 500–750 mg twice daily Levofloxacin (IV, oral) 500–750 mg daily Moxifloxacin (IV, oral) 400 mg daily	1 mo, 3 mo, then annually	1 mo, 3 mo, then annually		1 mo, 3 mo, then annually	Baseline			Consider more frequent monitoring (ie, weekly) with long courses and/or IV therapy Adjust dose for renal dysfunction (ciprofloxacin, levofloxacin) Close ECG monitoring with concomitant QTc-prolonging therapy
Imipenem-cilastatin (IV) [9, 10, 28] 500 mg–1 g every 8–12 h	Weekly	Weekly		Weekly				Adjust dose for renal dysfunction
Isoniazid (oral, IM) [9, 10, 15, 21, 31, 37] 5 mg/kg daily (max 300 mg)				Baseline, monthly				Administer with pyridoxine Hepatic panel may be reserved for patients with underlying liver disease or concomitant administration of hepatotoxic agents May consider TDM when indicated Peak (1–2 h): 3–6 mcg/mL
Macrolides [9, 10, 15, 22, 23, 31] Azithromycin (IV, oral) 250–500 mg daily or 500 mg 3 times/wk Clarithromycin (oral) 500 mg every 12 h	1 mo, 3 mo, then annually	1 mo, 3 mo, then annually		1 mo, 3 mo, then annually		Baseline		May consider TDM when indicated Azithromycin peak (2–3 h): 0.2–0.7 mcg/mL Clarithromycin peak (2–3 h): 2–7 mcg/mL Adjust dose for renal dysfunction (clarithromycin) Close ECG monitoring with concomitant QTc-prolonging therapy
Omadacycline (IV, oral) [9, 15, 38] 300 mg daily (oral) 100 mg daily (IV)	1 mo, 3 mo, then annually	1 mo, 3 mo, then annually		1 mo, 3 mo, then annually				Consider omission of loading dose for improved GI tolerability
Oxazolidinones [9, 10, 15, 39, 40] Linezolid (IV, oral) 300–600 mg daily Tedizolid (IV, oral) 200 mg daily	Weekly (biweekly if stable after 4 wk)		Consider trough level for linezolid	Baseline, monthly				Consider dose adjustments based on TDM for linezolid Trough (linezolid): 2–8 mcg/mL or <2 mcg/mL (long-term use)
Rifamycins [9, 10, 15, 24, 25, 31] Rifabutin (oral) 5 mg/kg daily (max 300 mg) 300 mg 3 times/wk Rifampin (IV, oral) 10 mg/kg daily (max 600 mg) 600 mg 3 times/wk	Baseline, 1 mo, then every 3 mo	Baseline, 1 mo, then every 3 mo		Baseline, 1 mo, then every 3 mo				May consider TDM when indicated Rifabutin peak (3 h): 0.45–0.9 mcg/mL Rifampin peak (2 h): 8–24 mcg/mL Reduce dose for renal dysfunction (rifabutin)
Trimethoprim-sulfamethoxazole (IV, oral) [9, 10, 15] • 160 mg/800 mg twice daily	1 mo, 3 mo, then annually	1 mo, 3 mo, then annually		1 mo, 3 mo, then annually				Consider weekly labs with higher doses and/or IV therapy Reduce dose for renal dysfunction
Tetracyclines [9, 10, 15] Doxycycline (IV, oral) • 100 mg twice daily Minocycline (IV, oral) • 100 mg twice daily	1 mo, 3 mo, then annually	1 mo, 3 mo, then annually		1 mo, 3 mo, then annually				
Tigecycline (IV) [9, 10, 28] • 25–50 mg every 12–24 h	Weekly	Weekly		Weekly				Reduce dose for liver dysfunction Consider omission of loading dose for improved GI tolerability

Abbreviations: BMP, basic metabolic panel; CBC, complete blood count; ECG, electrocardiogram; GI, gastrointestinal; IM, intramuscular; IV, intravenous; max, maximum; NTM, nontuberculous mycobacteria; TDM, therapeutic drug monitoring.

Although intravenous antibiotics may be unavoidable in some scenarios, vascular access device placement comes with associated risk and significant health care resources. Emerging oral therapies with broad-spectrum activity against rapid-growing mycobacteria, such as oxazolidinones and omadacycline, may provide an alternative to historically intravenous regimens in some patients [9]. Clinical data supporting these agents are limited, although ongoing clinical trials may clarify their role in therapy [9, 41–46]. Nonetheless, there remains an urgent need for additional, well-tolerated oral options with activity against species with limited susceptibility.

### Medication Acquisition

Clinicians are often tasked with procuring NTM medications, which can involve complex acquisition models. While Table 2 summarizes the general distribution models, a few unique scenarios are further described in this section.

**Table 2.** Outpatient NTM Medication Acquisition

Antimicrobial	FDA Indication for NTM	Pharmacy Availability	Prescription Requirements	Patient Acquisition
Amikacin (IV)	No	Vials for inhalation: Community Intravenous infusion: Home infusion or ITC	Vials for inhalation: Routine prescribing Intravenous infusion: Home infusion company forms	Vials for inhalation: Pharmacy pickup or home delivery Intravenous infusion: Home delivery
Amikacin (inhaled liposomal) [47, 48]	Yes	Specialty—2 pharmacies	Company-provided form or routine prescribing	Home delivery
Bedaquiline (oral) [49]	No	Specialty—1 pharmacy	Company-provided form	Clinic pickup
Cefoxitin (IV)	No	Home infusion or ITC	Home infusion company forms	Home delivery
Clofazimine (oral) [50–52]	Not FDA approved	Investigational	Drug company portal and research pharmacy protocol	Clinic pickup (home delivery may be possible)
Ethambutol (oral)	No	Community	Routine prescribing	Pharmacy pickup or home delivery
Fluoroquinolones (oral)	No	Community	Routine prescribing	Pharmacy pickup or home delivery
Imipenem-cilastatin (IV)	No	Home infusion or ITC	Home infusion company forms	Home delivery
Macrolides (oral)	No	Community	Routine prescribing	Pharmacy pickup or home delivery
Omadacycline (oral)	No	Community or Specialty	Routine prescribing	Pharmacy pickup or home delivery
Oxazolidinones (oral)	No	Community or Specialty	Routine prescribing	Pharmacy pickup or home delivery
Rifamycins (oral)	No^a^	Community	Routine prescribing	Pharmacy pickup or home delivery
Trimethoprim-sulfamethoxazole (oral)	No	Community	Routine prescribing	Pharmacy pickup or home delivery
Tetracyclines (oral)	No	Community	Routine prescribing	Pharmacy pickup or home delivery
Tigecycline (IV)	No	Home infusion or ITC	Home infusion company forms	Home delivery

Abbreviations: FDA, Food and Drug Administration; ITC, infusion therapy center; IV, intravenous; NTM, nontuberculous mycobacteria.

^a^Rifabutin has an FDA indication for the prevention of disseminated *M. avium* in patients with HIV.

With the exception of clofazimine, NTM medications are subject to prescription insurance coverage. As many are high cost, have niche indications, or are used off-label for NTM treatment, they may require prior authorization (PA) and/or appeals to be covered. Typically, the information required includes diagnosis (or International Classification of Diseases, 10th Revision, code), identified organism, and reasons why alternative therapies are not suitable. This may include allergies, drug interactions, or previous untoward effects. Susceptibility information can be important, especially if no other susceptible options are available. Referencing primary literature or national guidelines can improve PA approval rates, but some may still require a peer-to-peer discussion regarding the clinical case and need for a specific medication. Building electronic health record (EHR) templates may be helpful. A sample appeal letter can be found in Supplementary Figure 2.

Even with “covered” medications, patients may still face exorbitant copays. The drug manufacturer may have financial assistance programs or “copay cards” available. Additionally, philanthropic or research organizations may offer financial support. Two such organizations are The Assistance Fund [53] and HealthWell Foundation [54]. Programs vary based on what federal poverty level equivalent is eligible for assistance. Notably, Medicare recipients and other government insurances may be excluded from assistance. Institutions may have internal financial assistance programs, with more leniency in terms of federal poverty level. Programs can have different enrollment periods, limitations on financial aid provided, and annual capacities.

Inhaled liposomal amikacin is currently available via 2 specialty pharmacies. A combined prescription order and patient assistance form is available online [47], which can facilitate financial assistance and first-delivery in-home nurse teaching with the included inhalation device. The patient's signature is required to enroll in the teaching program. Insurance benefits investigation and PA can be completed by internal clinic staff or by the manufacturer via the enrollment form [48].

Bedaquiline is currently only available through 1 specialty pharmacy via a special ordering form that is faxed to the distributor. As the loading and maintenance dosing are vastly different, clinics can prepopulate a blank form and save for future use [49]. Once the copay is collected, medication is delivered to a medical clinic or office, and the patient should be contacted for pickup. The medication cannot be delivered to the patient's home.

Clofazimine is no longer commercially available in the United States but is available via expanded access under investigational drug access. If only prescribing for 1 patient, a Single Patient Investigational New Drug (SPIND) application can be submitted to the Food and Drug Administration (FDA) [50]. However, if multiple patient treatments are anticipated, clinicians are asked to initiate a multiple patient program at their institution [51]. Both avenues require coordination with the pharmaceutical company and the local institutional review board (IRB). Once approved, patient consent is obtained, and patients are individually enrolled via the manufacturer's online portal [52]. A patient-specific 90-day drug supply is requested through the portal by an ordering clinician listed on the IRB protocol and shipped to a research pharmacy. Clofazimine is provided free of charge (no insurance or copayment required), and patients pick up the medication from the clinic or research pharmacy. Since the coronavirus disease 2019 pandemic, some states may allow research pharmacies to mail the medication to the patient.

Medications with limited distribution models and/or dispensing via specialty pharmacies may not appear in third-party refill records. Tasking a team member with maintaining a tracking document to ensure timely refills and address insurance issues may facilitate improved oversight and adherence to therapy.

### Therapeutic Drug Monitoring

Analogous to other infectious diseases, TDM in the treatment of NTM is valuable when relationships between exposures and efficacy or toxicity have been established, therapies demonstrate pharmacokinetic variability, patient organ function changes, patients take interacting medications, barriers to adherence exist, disease prognosis is guarded, or immunologic defects are persistent and/or severe [55].

A key driver of TDM is the establishment of exposure–response relationships with efficacy or toxicity. Pharmacokinetic/pharmacodynamic (PK/PD) efficacy targets are frequently established through in vitro data and infection models and/or retrospective analyses of clinical data [55]. While much progress has been made in recent years using in vitro models such as the hollow-fiber model, retrospective and prospective clinical validation remains limited for most NTM therapies.

Relationships between exposures and associated toxicities play a particularly important role in NTM treatment due to the extended duration of therapy required, variable tolerability, occasional overlapping toxicities, and limited therapeutic options. Furthermore, physical characteristics of body composition, such as cachexia, may impact drug pharmacokinetics [56]. Thus, TDM may be used to minimize the impact of toxicities on regimen selection, adherence, and patient outcomes.

In clinical practice, routine TDM is standard of care for patients receiving intravenous amikacin for NTM given the risk of serious adverse events, notably nephrotoxicity and ototoxicity. A calculated maximum concentration (C_max_) is recommended, ideally extrapolated using serum drug levels collected at 2 and 6 hours following administration to avoid sampling during the redistribution phase. The target C_max_ for intravenous amikacin is 35–45 mcg/mL (15-mg/kg dose) or 65–80 mcg/mL (25-mg/kg dose), while the target trough should be undetectable [15]. TDM for additional antimycobacterials should be considered in clinical scenarios with concern for low drug exposure, including patients with inadequate clinical or microbiological response [10]. Drug exposure may be compromised in the setting of malabsorption (bariatric surgery, severe gastrointestinal disease) and drug interactions, which could lead to treatment failure and/or development of resistance [15]. A comprehensive review of the data supporting the PK/PD targets of antimycobacterials was recently compiled [57], and C_max_ targets for select oral agents are included in Table 1 [31].

Implementation of TDM in the clinic is complicated for several reasons. First, very few US laboratories possess clinically validated antimycobacterial drug assays. Thus, a reference laboratory must be identified and workflows established. This requires substantial coordination between the multidisciplinary team, the facility's laboratory, and the external laboratory to ensure that TDM is successfully conducted. Once a reliable clinical assay is available, the TDM approach and interpretation add another layer of complexity. Although antimycobacterial TDM has historically focused primarily on the determination of peak or trough concentrations, growing evidence suggests that population PK and Bayesian dosing adjustments may play a greater role in optimizing antimycobacterial regimens, similar to tuberculosis [57, 58]. While these approaches have the potential to give patients the best chance at clinical success through individualized drug therapy, use of the required software, result interpretation, and dose adjustments require additional resources and expertise. Despite logistical challenges, antimycobacterial TDM using a reference laboratory for testing has been clinically implemented in at least 1 large academic medical center in the United States [59]. Referral laboratories for antimycobacterial TDM include University of Florida-Shands and National Jewish, and antimycobacterial TDM is in exploration at Mayo Clinic [20–25, 38].

### Adverse Drug Reactions

Managing NTM infections often involves managing ADRs. Common and notable ADRs with mitigation strategies, management recommendations, and additional considerations are listed in Table 3.

**Table 3.** Notable Adverse Drug Reactions With NTM Therapies

Antimicrobial	Notable Adverse Drug Reactions^a^	Mitigation Strategies	Comments
Amikacin (IV) [9, 60]	• Nephrotoxicity • Ototoxicity	Routine TDM Consider thrice-weekly dosing interval for improved safety Periodic audiology exams	Ototoxicity generally irreversible
Amikacin (inhaled liposomal) [9, 32]	• Dysphonia (48%) • Bronchospasm (29%) • Sore throat (18%) • Hemoptysis (18%) • Ototoxicity (17%)	Periodic audiology exams	Ototoxicity generally irreversible
Bedaquiline [9, 33, 61, 62]	• QTc prolongation • Gastrointestinal symptoms (38%) • Arthralgia (33%) • Increased serum transaminases (9%) • Hepatotoxicity (rare)	Routine liver function safety monitoring (AST, ALT, bilirubin, INR, PT) ECG monitoring and close repletion of magnesium and potassium, especially with concomitant QTc-prolonging medications Administer with food to increase absorption and potentially mitigate gastrointestinal symptoms	Consider discontinuation if meets an FDA guidance threshold for hepatotoxicity^b^
Cefoxitin [9, 34]	• Rash, allergic reactions • Gastrointestinal symptoms • Myelosuppression (rare)		
Clofazimine [9, 35, 62]	• Discoloration of skin and/or body fluids (75%–100%) • QTc prolongation • Gastrointestinal symptoms (40%–50%)	Counsel patients to avoid prolonged direct sun exposure and utilize strong sunscreens to avoid exacerbating skin effects ECG monitoring and close repletion of magnesium and potassium, especially with concomitant QTc-prolonging medications Administer with food to increase absorption and potentially mitigate gastrointestinal symptoms Start with lower dose (50 mg daily) for 1–2 wk if GI intolerance	Carefully weigh risks and benefits of therapy with patient, as dermatologic reactions can lead to serious psychological effects, including depression and suicide Skin discoloration may take months to years to resolve following therapy discontinuation
Ethambutol [9, 36]	• Optic neuritis • Peripheral neuropathy (rare) • Color vision changes • Gastrointestinal symptoms	Consider ophthalmology consult if history of vision problems Ishihara color vision test at each clinic visit Administer with food to potentially mitigate gastrointestinal symptoms	Discontinuation is recommended if vision changes occur; incomplete recovery and even blindness have been reported
Fluoroquinolones [9, 62–65]	• QTc prolongation • Tendonitis (rare) • *Clostridioides difficile* • Hypoglycemia	ECG monitoring and close repletion of magnesium and potassium, especially with concomitant QTc-prolonging medications	
Imipenem-cilastatin [9, 61, 66]	• Seizures (0.4%) • Hepatotoxicity (rare) • Myelosuppression (rare)	Routine liver function safety monitoring (AST, ALT, bilirubin, INR, PT)	Consider discontinuation if meets an FDA guidance threshold for hepatotoxicity^b^
Macrolides [9, 10, 61, 62, 67, 68]	• QTc prolongation • Gastrointestinal symptoms (1%–9%) • Photosensitivity • Ototoxicity, tinnitus (long-term use) • Hepatotoxicity (rare)	Routine liver function safety monitoring (AST, ALT, bilirubin, INR, PT) ECG monitoring and close repletion of magnesium and potassium, especially with concomitant QTc-prolonging medications Administer with food to potentially mitigate gastrointestinal symptoms	Consider discontinuation if meets an FDA guidance threshold for hepatotoxicity^b^ Ototoxicity, tinnitus generally reversible within a month of discontinuation Azithromycin generally preferred over clarithromycin given improved tolerability, once-daily dosing, and fewer drug interactions
Omadacycline [9, 61, 69]	• Gastrointestinal symptoms (2%–22%) • Increased serum transaminases (2%–4%) • Hepatotoxicity (rare)	Routine liver function safety monitoring (AST, ALT, bilirubin, INR, PT) Consider omission of loading dose for improved GI tolerability	Consider discontinuation if meets an FDA guidance threshold for hepatotoxicity^b^ Administer on strict empty stomach for optimal absorption
Oxazolidinones [9, 39, 40, 70, 71]	• Myelosuppression • Peripheral neuropathy	Administer linezolid as once-daily dosing to minimize myelosuppression risk Consider linezolid TDM to mitigate hematologic toxicity	Discontinuation is recommended if vision changes occur; incomplete recovery and even blindness have been reported
Rifamycins [9, 61, 72, 73]	• Discoloration of body fluids • Hepatotoxicity • Gastrointestinal symptoms • Myelosuppression	Routine liver function safety monitoring (AST, ALT, bilirubin, INR, PT) Perform medication reconciliation and drug interaction screen at each visit	Consider discontinuation if meets an FDA guidance threshold for hepatotoxicity^b^
Trimethoprim-sulfamethoxazole [9, 74, 75]	• Gastrointestinal symptoms • Rash • Nephrotoxicity • Hyperkalemia • Myelosuppression	Perform medication reconciliation and drug interaction screen at each visit	Mild pseudo-elevation in serum creatinine may occur; significant elevations may indicate interstitial nephritis or acute tubular necrosis
Tetracyclines [9, 61, 76, 77]	• Photosensitivity • Esophageal ulceration • Gastrointestinal symptoms • Drug-induced lupus (minocycline) • Hepatotoxicity (rare) • Hyperpigmentation (long-term use)	Routine liver function safety monitoring (AST, ALT, bilirubin, INR, PT) Administer with food to potentially mitigate gastrointestinal symptoms	Consider discontinuation if meets an FDA guidance threshold for hepatotoxicity^b^ Blue-gray hyperpigmentation reported with long-term minocycline and doxycycline use
Tigecycline [9, 78, 79]	• Gastrointestinal symptoms (12%–35%) • Increased serum transaminases (3%–5%) • Hepatotoxicity (rare)	Routine liver function safety monitoring (AST, ALT, bilirubin, INR, PT) Dose-limiting GI toxicity; consider omission of loading dose and titration starting at 12.5–25 mg IV daily with target dose 50 mg IV every 12 h as tolerated	Consider discontinuation if meets an FDA guidance threshold for hepatotoxicity^b^

Abbreviations: AST, aspartate aminotransferase; ALT, alanine aminotransferase; ECG, electrocardiogram; FDA, Food and Drug Administration; GI, gastrointestinal; INR, international normalized ratio; NTM, nontuberculous mycobacteria; PT, prothrombin time; TDM, therapeutic drug monitoring; ULN, upper limit of normal.

^a^Incidence rates where noted come from the applicable product labeling as of October 30, 2023.

^b^ALT or AST >8× the upper limit of normal (ULN); ALT or AST >5×ULN for >2 weeks; ALT or AST >3×ULN and (total bilirubin >2×ULN or INR >1.5); ALT or AST >3×ULN with the appearance of fatigue, nausea, vomiting, right upper quadrant pain or tenderness, fever, rash, and/or eosinophilia.

The most frequently encountered ADRs involve the gastrointestinal (GI) tract and may occur with any NTM-active therapy. Effects include diarrhea, nausea, vomiting, abdominal pain, and/or dysgeusia. Hepatotoxicity has been associated most notably with rifamycins, bedaquiline, tetracyclines, imipenem-cilastatin, and macrolides. While not evaluated systematically, the FDA provides guidance on therapy discontinuation when liver injury occurs in clinical trials (Table 3 legend) [61]. Nephrotoxicity has been causally linked to trimethoprim-sulfamethoxazole and is well documented with aminoglycoside use. Trimethoprim inhibits cellular transport proteins in the proximal tubule, responsible for serum creatinine secretion, leading to a mild pseudo-elevation of creatinine within hours of medication initiation [75]. However, trimethoprim-sulfamethoxazole can also rarely lead to acute interstitial nephritis or acute tubular necrosis. Ototoxicity (primarily cochlear toxicity) with long-term intravenous or inhaled amikacin is primarily irreversible [80]; however, hearing loss or tinnitus is generally reversible within a month of treatment discontinuation with macrolide therapy [81]. Ethambutol or oxazolidinones may cause optic neuritis and/or peripheral neuropathy, which tend to be duration-dependent, generally occurring after at least 1 month of therapy [82]. Oxazolidinones can also result in myelosuppression with prolonged use, generally thrombocytopenia and/or anemia, more so with linezolid than tedizolid [39, 83]. Trimethoprim-sulfamethoxazole, rifamycins, and β-lactams have also been associated with hematologic toxicity [9, 84]. Treatment modification should be considered with rapid decreases in hemoglobin/hematocrit, neutrophils, or platelets while on therapy in those without an alternative etiology for myelosuppression. Idiosyncratic dermatologic and cosmetic ADRs may occur. Rifamycins are associated with a benign orange discoloration of body fluids, including sweat, saliva, urine, and tears [9]. Similarly, clofazimine can cause an orange-brown discoloration of body fluids and, much more commonly, the skin [35]. Tetracyclines also carry photosensitivity precautions. Blue-gray skin discoloration may also occur with long-term use, particularly with minocycline.

Given the polypharmacy nature of treating NTM, patients are often at increased risk of ADRs when multiple agents with similar risks of toxicity are used in combination. Some centers may use a phased-in approach (eg, stagger medication starts on a weekly basis) to assess for adverse reactions and aim to improve tolerability. Additionally, lower doses may be used for a week or 2 before progressing to the full dose. Clinicians are encouraged to report ADRs to the FDA Adverse Event Reporting System (FAERS) via MedWatch to aid in postmarketing surveillance efforts [85].

### Drug–Drug Interactions

Drug interactions with NTM therapy most commonly occur by 1 of 3 mechanisms: (1) metabolic interactions, usually involving cytochrome P450 (CYP) isoenzymes or transmembrane transporters like P-glycoprotein (P-gp), (2) additive or overlapping toxicity, or (3) diminished oral absorption due to chelation by concomitantly administered multivalent cation-containing compounds, such as calcium, magnesium, iron, aluminum, and zinc. In addition, elimination of vitamin K–producing bacteria by antimicrobials may contribute to alterations in coagulation for patients taking warfarin. Comprehensive references and interaction databases should be consulted when providing patient care [4]; Table 4 provides a nonexhaustive summary of relevant drug interactions. Given the potential for discrepancies and variable performance in interaction identification and severity classification among databases, expert review by a clinical pharmacist can aid in the interpretation, mitigation, and management of drug interactions [86].

**Table 4.** Notable Drug Interactions With NTM Therapies

Antimicrobial	Metabolism	Effect on Drug Metabolism	Potential Interacting Agent(s)	Comments
Amikacin [60]	None known		Nephrotoxic medications	Aminoglycosides may enhance nondepolarizing neuromuscular blocker effects Concomitant loop diuretic therapy may increase risk of nephrotoxicity, ototoxicity
Azithromycin [67, 68, 87]	Substrate: CYP3A4 (minor)	Inhibition: CYP3A4 (weak) P-gp	QTc-prolonging agents Warfarin	Less interaction potential compared with clarithromycin
Bedaquiline [33]	Substrate: CYP3A4 (major) CYP2C19 (minor) CYP2C8 (minor)		CYP3A4 inhibitors/inducers QTc-prolonging agents	Avoid use with strong CYP3A4 inducers Avoid use with ≥14 d of strong CYP3A4 inhibitors
Cefoxitin [34]	Substrate: OAT1/3			Low interaction potential
Ciprofloxacin [63, 88–91]	Substrate: OAT1/3 P-gp	Inhibition: CYP1A2 (moderate) CYP3A4 (weak)	CYP1A2 substrates Oral hypoglycemic agents Multivalent cations (oral administration) QTc-prolonging agents Warfarin	Administer (oral) 2 h before or 6 h after multivalent cations CYP1A2-medicated interactions (tizanidine, theophylline, etc.) unique to ciprofloxacin among contemporary fluoroquinolones
Clarithromycin [68]	Substrate: CYP3A4 (major)	Inhibition: CYP3A4 (strong) OATP1B1 P-gp	CYP3A4 substrates, inhibitors, inducers QTc-prolonging agents Warfarin	Higher interaction potential compared with azithromycin
Clofazimine [35, 92]	None known	Inhibition: CYP3A4 CYP2C8 (moderate) CYP2D6 (moderate)	CYP2C8, 2C9, 3A4 substrates QTc-prolonging agents	
Ethambutol [36]	Substrate: OCT1, OCT2			Low interaction potential
Imipenem-cilastatin [66]	Substrate: Dehydropeptidase-1 (imipenem)		Valproic acid	
Isoniazid [37, 93]	Substrate: CYP2E1 (minor)	Inhibition: CYP2E1 (weak) CYP2C19 (moderate) CYP3A4 (moderate) Induction: CYP2E1 (weak)	Hepatotoxic agents CYP2C19 substrates	Genetic variability in N-acetyltransferase 2 (NAT2) may predispose to increased drug interaction potential; slow acetylators will experience decreased drug clearance and higher concentrations
Levofloxacin [64, 88–91]	Substrate: OAT1/3		Oral hypoglycemic agents Multivalent cations (oral administration) QTc-prolonging agents Warfarin	Administer (oral) 2 h before or 2 h after multivalent cations
Linezolid [70, 94, 95]	None known	Inhibition: MAO (weak)	Adrenergic agents Serotonergic agents	Contemporary data suggest low risk of adverse events when linezolid used concomitantly with serotonergic agents
Moxifloxacin [65, 88–91]	None known		Oral hypoglycemic agents Multivalent cations (oral administration) QTc-prolonging agents Warfarin	Administer (oral) 4 h before or 8 h after multivalent cations
Rifabutin [73, 96]	Substrate: CYP3A4 (major) CYP1A2 (minor)	Induction: CYP2C9 (weak) CYP3A4 (moderate)	CYP3A4 substrates, inhibitors, inducers Hepatotoxic agents Thyroid products	Monitor TSH closely when administered with thyroid products
Rifampin [72, 96]	Substrate: OATP1B1/1B3 P-gp	Induction: CYP2B6 (moderate) CYP2C19 (strong) CYP2C8 (moderate) CYP2C9 (moderate) CYP3A4 (strong) OATP1B1/1B3 P-gp UGT1A1	CYP2C19, 3A4 substrates Hepatotoxic agents Thyroid products	Induction potential rifampin > rifabutin High potential for drug interactions; screen medications closely Monitor TSH closely when administered with thyroid products
Tedizolid [71, 97]	None known	Inhibition: BCRP	Serotonergic agents Adrenergic agents	Appears to have less serotonergic interaction potential compared with linezolid
Tetracyclines [69, 76, 77]	None known		Multivalent cations (oral administration)	Separate oral administration with multivalent cations by several hours
Trimethoprim-sulfamethoxazole [74, 98, 99]	Substrate: CYP2C9 (sulfamethoxazole)	Inhibition: CYP2C9 (moderate) CYP2C8 (strong) OCT2	Warfarin Oral hypoglycemic agents ACE-I, ARB Phenytoin Potassium-sparing diuretics	Caution of additive hyperkalemic effect when combined with potassium-sparing agents

Abbreviations: ACE-I, angiotensin converting enzyme inhibitor; ARB, angiotensin receptor blocker; BCRP, breast cancer resistance protein; CYP, cytochrome P450; MAO, monoamine oxidase; NTM, nontuberculous mycobacteria; OAT, organic anion transporter; OATP, organic anion transporting polypeptides; OCT, octamer-binding protein; P-gp, P-glycoprotein; TSH, thyroid-stimulating hormone; UGT, UDP-glucuronosyltransferase.

Interaction potential can be influenced by dosage, duration, inhibition or induction potency, and metabolic enzyme binding affinity. Most metabolic interactions are manifested through the CYP system of enzymes. CYP inducers can increase metabolism of CYP substrates (resulting in decreased substrate levels), while CYP inhibitors can decrease metabolism of CYP substrates (resulting in increased substrate levels). Thousands of medications undergo some degree of metabolism by the CYP enzymes. CYP3A4/5 is the most predominant in humans and is responsible for many interactions. Medications subject to CYP-based interactions are maintained on the FDA's website [100].

An additional type of metabolic interaction involves the use of linezolid with serotonergic agents. Linezolid is a weak and reversible inhibitor of monoamine oxidase (MAO). This inhibition blocks oxidative deamination to cause the accumulation of endogenous catecholamines (serotonin and norepinephrine). Combining linezolid with serotonergic or adrenergic agents, or tyramine-containing foods, can lead to increased blood pressure or the rare occurrence of serotonin syndrome [94, 95]. Of note, contemporary data suggest an incidence of linezolid-associated serotonin syndrome well below 1%, even when co-administered alongside other agents with serotonergic activity [94]. Tedizolid is a newer oral oxazolidinone believed to have an even lower risk of these interactions due to its weaker MAO inhibition and lower central nervous system penetration [97].

Organ toxicity may also occur. QTc prolongation can increase the risk of ventricular arrhythmias. Many medications can prolong QTc, and the risk of arrhythmia can be increased by combining these agents. Among NTM medications, fluoroquinolones, macrolides, bedaquiline, and clofazimine have all been reported to prolong QTc intervals. Electrocardiography and electrolyte monitoring are recommended, particularly when these agents are used in combination, and close repletion of potassium and magnesium may be indicated to mitigate toxicity [62]. Combining NTM agents with hepatotoxic potential may increase the risk of an adverse event [101]. Likewise, patients could experience higher nephrotoxicity rates when multiple nephrotoxic agents are combined [102].

Additional miscellaneous interactions exist. Trimethoprim has been associated with an antikaliuretic effect, which can contribute to a significant hyperkalemic response when administered with potassium-sparing agents like angiotensin-converting enzyme inhibitors, angiotensin receptor blockers, or spironolactone [98, 99]. Fluoroquinolones can cause hypoglycemia by increasing insulin release via blockade of adenosine triphosphate-sensitive potassium channels in pancreatic β cells [90]. This effect can be accentuated in patients taking oral hypoglycemic therapy. Orally administered fluoroquinolones and tetracycline analogs can have their absorption diminished via chelation, when concomitantly administered with multivalent cations. Patients should be advised to separate administration of these agents from any multivalent cation to ensure adequate absorption; specific time intervals for separation are listed in Table 4 [63–65, 69, 76, 77, 91, 103]. Trimethoprim-sulfamethoxazole interacts with methotrexate through anion transport inhibition, which leads to an additive inhibition of dihydrofolate reductase [98]. This interaction can contribute to pancytopenia.

### Multidisciplinary NTM Care Team

A multidisciplinary team approach is ideal in the management of NTM infection. It may be said that NTM care “takes a village.” The NTM care team encompasses several disciplines, including but not limited to pulmonology, infectious diseases, radiology, microbiology, respiratory therapy, primary care, and pathology (Figure 1) [104]. Diagnosis of NTM disease is complex, at times taking as long as 20 months, and requires communication and coordination among pulmonologists, infectious disease specialists, radiologists, and clinical microbiologists [105]. Disease management may additionally involve respiratory therapists, surgeons, nurses, and/or dieticians [106]. Clinical pharmacists specializing in NTM can assist with antimycobacterial dosing and TDM, ADR management, and drug interaction mitigation; guidance on implementing a pharmacist resource for NTM is available in Supplementary Table 1 [59]. Social workers and patient support groups can be incorporated to help patients cope with psychosocial and financial concerns [106]. Clinical outcomes research with the multidisciplinary team may be further supportive of this approach.

**Figure 1.**
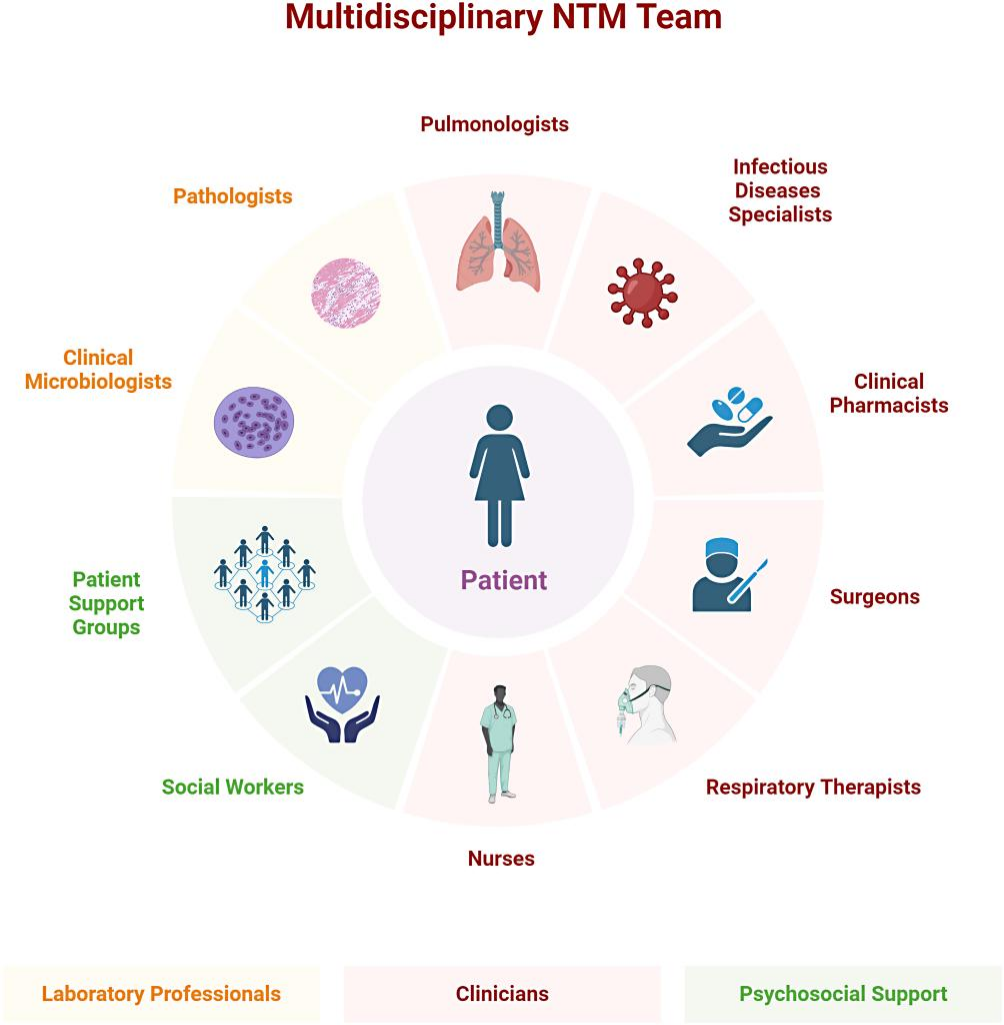
Multidisciplinary NTM care team. Created with BioRender.com. Abbreviation: NTM, nontuberculous mycobacteria.

## CONCLUSIONS

NTM infections are increasing in prevalence, and their management involves highly complex treatment and monitoring considerations. Effective treatment of NTM requires polypharmacy, often based on interpretation of unvalidated susceptibility breakpoints. Emerging evidence suggests a role for TDM in this patient population, and intimate knowledge is required of drug interactions, adverse effect management, pharmaceutical acquisition, and financial assistance. A multidisciplinary care team is ideal for the care of NTM patients, with a growing role for clinical pharmacist involvement.

## Supplementary Material

ofae128_Supplementary_Data

## Supplementary Data

Supplementary materials are available at *Open Forum Infectious Diseases* online. Consisting of data provided by the authors to benefit the reader, the posted materials are not copyedited and are the sole responsibility of the authors, so questions or comments should be addressed to the corresponding author.
