# Supplementary material for: Pharmacotherapeutic Considerations in the Treatment of Nontuberculous Mycobacterial Infections: A Primer for Clinicians
Source: Open Forum Infect Dis. 2024 Mar 15;11(4):ofae128. doi: 10.1093/ofid/ofae128 (PMC10977864; doi:10.1093/ofid/ofae128)
Supplement: ofae128_Supplementary_Data [file ofae128_supplementary_data.zip › Cimino NTM OFID Supplemental Figure 1.docx]

Supplemental Figure 1: Example susceptibility report

Organism: *Mycobacterium abscessus complex*

Source: Sputum

| **Antibiotic** | **MIC (mcg/mL)** | **S** | **I** | **R** |
| --- | --- | --- | --- | --- |
| Amikacin | 16 | ✓ |  |  |
| Bedaquiline | 0.12 |  |  |  |
| Cefoxitin | 32 |  | ✓ |  |
| Clarithromycin^1^ | 1 | ✓ |  |  |
| Clofazimine | 0.12 |  |  |  |
| Ciprofloxacin^2^ | > 4 |  |  | ✓ |
| Doxycycline | > 16 |  |  | ✓ |
| Eravacycline | ≤ 0.06 |  |  |  |
| Ertapenem | - |  |  |  |
| Imipenem | 16 |  | ✓ |  |
| Linezolid | 8 | ✓ |  |  |
| Meropenem | - |  |  |  |
| Moxifloxacin | > 8 |  |  | ✓ |
| Minocycline | > 8 |  |  | ✓ |
| Omadacycline | 0.25 |  |  |  |
| Tedizolid | ≤ 0.5 |  |  |  |
| Tigecycline | 0.03 |  |  |  |
| Tobramycin | - |  |  |  |
| Trimethoprim-sulfamethoxazole | 8/152 |  |  | ✓ |

Abbreviations: MIC, minimum inhibitory concentration; S, susceptible; I, intermediate; R, resistant

Footnotes:

^1^Clarithromycin incubated for 14 days to test for inducible resistance (erm gene). Azithromycin susceptibility can be interpreted from clarithromycin.

^2^Ciprofloxacin and levofloxacin are interchangeable. Both are less active *in vitro* compared to moxifloxacin.

There are no established interpretive guidelines (breakpoints) for agents reported without interpretation.

The above was created by authors and does not contain patient information
