# Supplementary material for: Pharmacotherapeutic Considerations in the Treatment of Nontuberculous Mycobacterial Infections: A Primer for Clinicians
Source: Open Forum Infect Dis. 2024 Mar 15;11(4):ofae128. doi: 10.1093/ofid/ofae128 (PMC10977864; doi:10.1093/ofid/ofae128)
Supplement: ofae128_Supplementary_Data [file ofae128_supplementary_data.zip › Cimino NTM OFID Supplemental Figure 2.docx]

Supplemental Figure 2: Sample insurance appeal letter

[DATE]

To Whom This May Concern

RE: [PATIENT NAME] [DOB]

[INSURANCE POLICY NUMBER (IF KNOWN)]

Dear Sir/Madam:

[PATIENT NAME] is a [XX] year old patient with a non-tuberculous mycobacterial pulmonary infection. Cultures obtained on [DATE] grew Mycobacterium [SPECIES] with the following susceptibility profile:

[COPY/PASTE SUSCEPTIBILITY PROFILE OR SUMMARIZE]

Resistant to: [ANTIBIOTIC 1], [ANTIBIOTIC 2], and [ANTIBIOTIC 3]

Intermediate to: [ANTIBIOTIC 4] and [ANTIBIOTIC 5]

Susceptible to: [ANTIBIOTIC 6], [ANTIBIOTIC 7], [ANTIBIOTIC 8], and [ANTIBIOTIC 9]

Additional pertinent laboratory information:

[CAN PROVIDE LABORATORY VALUES & DATES AS NEEDED]

- [CBC]
- [Scr/BUN]
- [LFT]
- [QTc]
- Additional relevant values

Additional pertinent medications:

[CAN PROVIDE INTERACTING/RELEVANT MEDICATION LISTS HERE]

- [MEDICATION 1] and [DOSE]
- [MEDICATION 2] and [DOSE]

Per the 2020 IDSA Nontuberculous Mycobacterial guidelines, the treatment for M. [SPECIES] is a 3-drug regimen. The treatment duration is at least [12 months after sputum culture conversion] or [ALTERNATIVE DURATION].

Citation: <https://pubmed.ncbi.nlm.nih.gov/32797222/>

[PATIENT NAME] was started/will be started on [ANTIBIOTIC 6] at [DOSE] and [ANTIBIOTIC 7] at [DOSE]. [ANTIBIOTIC 8] cannot be utilized in [PATIENT NAME] because of:

- [ALLERGY INFORMATION]
- [DRUG INTERACTION INFORMATION]
- [PREVIOUS USE AND UNTOWARD EFFECTS]
- [OTHER PERTINENT INFORMATION]

Therefore, in order to follow national treatment guidelines, based on susceptibility information, and [REASON STATED ABOVE], the only other treatment option for [PATIENT NAME] is [ANTIBIOTIC 9] at [DOSE]. Please approve [ANTIBIOTIC 9] for this patient.

**Optional Information as Needed**

[ANTIBIOTIC 9] has been used in the treatment of M. [SPECIES] as evident by the following citations:

- [PUBMED CITATION 1]
- [PUBMED CITATION 2]

Sincerely,

[NAME] [CREDENTIALS]

[CLINIC NAME & INFORMATION]

[PHONE NUMBER]

[EMAIL]
