# Supplementary material for: Pharmacotherapeutic Considerations in the Treatment of Nontuberculous Mycobacterial Infections: A Primer for Clinicians
Source: Open Forum Infect Dis. 2024 Mar 15;11(4):ofae128. doi: 10.1093/ofid/ofae128 (PMC10977864; doi:10.1093/ofid/ofae128)
Supplement: ofae128_Supplementary_Data [file ofae128_supplementary_data.zip › Cimino NTM OFID Supplemental Table 1.docx]

Supplemental Table 1. Guidance on integrating clinical pharmacists into NTM care

| Practice Leadership Steps | Details |
| --- | --- |
| 1. Role Justification | - Develop a business plan that justifies the role to hospital leadership - Persuasive themes:   - High cost of NTM admissions   - Increasing incidence of NTM disease   - Complexity of NTM care   - Prevalent polypharmacy |
| 1. Training | - Recruit clinicians with existing experience in Infectious Diseases, OPAT, Ambulatory Care, or Pulmonology - Shadowing other NTM clinicians - In-services and webinars on NTM - NTM training checklists - NTM literature assignments |
| 1. Referral structure | - Raise awareness of the new service line with referring clinicians through institutional meetings and emails, establish physician proponent(s) - Create consult order for NTM pharmacist in EHR as face-to-face, video, and phone - Embed the order in referring specialty order panels and order sets |
| 1. Licensure extensions | - Develop and implement a collaborative practice agreement which allows pharmacist to:   - Adjust antimicrobial doses   - Order monitoring labs and procedures (i.e., ECGs)   - Order supportive care medications (i.e., ondansetron)   - Change antimicrobial(s) after discussion with collaborating physician |
| 1. Billing | Pharmacist can use MTM billing codes and incident-to-physician billing |

Abbreviations: ECG, electrocardiogram; EHR, electronic health record; MTM, medication therapy management; NTM, nontuberculous mycobacterium; OPAT, outpatient parenteral antimicrobial therapy
